# Identification of Anti-HIV Biomarkers of *Helichrysum* Species by NMR-Based Metabolomic Analysis

**DOI:** 10.3389/fphar.2022.904231

**Published:** 2022-07-22

**Authors:** Simin Emamzadeh Yazdi, Heino Martin Heyman, Gerhard Prinsloo, Thomas Klimkait, Jacobus Johannes Marion Meyer

**Affiliations:** ^1^ Department of Plant and Soil Sciences, University of Pretoria, Pretoria, South Africa; ^2^ Metabolon Inc., Morrisville, NC, United States; ^3^ Department of Agriculture and Animal Health, University of South Africa (UNISA), Pretoria, South Africa; ^4^ Molecular Virology, Department of Biomedicine, University of Basel, Basel, Switzerland

**Keywords:** biomarker, chlorogenic acids, *Helichrysum*, human immunodeficiency virus, metabolomics, quinic acid

## Abstract

Several species of the *Helichrysum* genus have been used ethnobotanically to treat conditions that we today know have been caused by viral infections. Since HIV is a modern disease with no ethnobotanical history, we commenced with a study on the anti-HIV activity of several *Helichrysum* species. Drug discovery of small molecules from natural resources that is based on the integration of chemical and biological activity by means of metabolomical analyses, enables faster and a more cost-effective path to identify active compounds without the need for a long process of bioassay-guided fractionation. This study used metabolomics to identify anti-HIV compounds as biomarkers from 57 *Helichrysum* species in a combined study of the chemical and biological data of two previous studies. In the OPLS-DA and hierarchical cluster analyses, anti-HIV activity data was included as a secondary observation, which assisted in the correlation of the phytochemical composition and biological activity of the samples. Clear grouping revealed similarity in chemical composition and bioactivity of the samples. Based on the biological activity of polar extracts, there was a distinct phytochemical difference between active and non-active groups of extracts. This NMR-based metabolomic investigation showed that the chlorogenic acids, compounds with cinnamoyl functional groups, and quinic acid were the most prominent compounds in the *Helichrysum* species with anti-HIV activity. This study further revealed that the chlorogenic acid type compounds and quinic acid are biomarkers for anti-HIV activity.

## Introduction

Medicinal plants treated several diseases throughout the history of mankind and it led to many investigations to identify the metabolites responsible for their curative effects. These bioactive compounds have been the source of many ‘modern’ pharmaceutical drugs (Satheeshkumar et al., 2012; Wang et al., 2017).

The selection of specific biomarkers for bioactivity from thousands of metabolites in medicinal plants has been a difficult challenge (Wang et al., 2017). Due to the variable sources and chemical complexity of medicinal plants, the use of only chromatographic techniques to find bioactive compounds and to standardize botanical extracts, has limitations. DNA-based molecular markers have usually precedent in fields such as taxonomy, physiology, genetics, medicine, etc. to identify biomarkers (Joshi et al., 2004; Pourmohammad, 2013). In contrast, identifying the secondary metabolite biomarkers using NMR-based metabolomic analysis has emerged as a new technique (Markley et al., 2017; Jahangir, et al., 2018).

Some secondary metabolites play important roles in immune function enhancement and exhibit antiviral potential, including against the Human Immunodeficiency Virus (HIV) (Salehi et al., 2018). HIV has infected around 75 million people worldwide. According to the World Health Organization (WHO), approximately 38 million are living with the infection with most of the infected population in sub-Saharan Africa (Deeks et al., 2015; World Health Organization, 2020).

Metabolomic studies of plants are of great importance when one wants to associate bioactivity with the chemical composition of the extract (Castro et al., 2020) and also to identify biomarkers employing metabolic fingerprinting and profiling (Madsen et al., 2010; Takayama et al., 2015). The techniques, ^1^H NMR spectroscopy and multivariate data analysis, are complimentary for studying the biochemical composition and metabolic pathways for discovering biomarkers from natural resources such as medicinal plants (Satheeshkumar et al., 2012). Principal component analysis (PCA) and orthogonal projections to latent structures discriminant analysis (OPLS-DA) can classify chemical groups with particular bioactivities and also identify the components responsible for the groupings which could well be used as biomarkers (Castro et al., 2020).

The *Helichrysum* genus of the Asteraceae family, is widely recognised for its many traditional medicinal plants used for the treatment of several medical conditions like nervousness and hysteria, and also to treat wounds, bacterial and viral infections and respiratory conditions (Meyer et al., 1996; Lourens et al., 2004; Lourens et al., 2008; Van Vuuren, 2008). It consists of approximately 500–600 species of which 245 are indigenous to southern Africa including Namibia (Lourens et al., 2004; Lourens et al., 2008). This genus has been the source of many interesting and bioactive compounds (Lourens et al., 2004; Appendino et al., 2007; Bauer et al., 2011; Kutluk et al., 2018). The extracts and the essential oils from this species have exhibited promising biological activities in various *in vitro* assays, which include anti-oxidant, antimicrobial, antifungal, anti-inflammatory and antiviral activity (Van Vuuren, 2008; Akaberi et al., 2019; Akinyede et al., 2021). In case of antiviral activity of *Helichrysum* genus there are several reports. According to Viegas et al. (2014), flavonoids and phloroglucinols isolated from *H. italicum* has inhibition activity against herpes simplex virus 1 (HSV1) and HIV, respectively. Most interesting though are the findings by Appendino et al. (2007) that arzanol (a phloroglucinol α–pyrone) inhibits HIV-1 replication in T-cells and inhibited NF-ҡB (IC_50_ = 5 μg/ml) indicating that this group of compounds may exhibit both antiviral and anti-inflammatory properties. The ethanol extract of *H. cymocum* exhibited the virucidal activity against the HSV1, the measles virus strain Edmonston A (MV-EA) as well as the Semliki forest virus A7 (Sindambiwe et al., 1999). Aqueous extracts of *H. aureonitens* exhibited antiviral activity against the HSV1 *in vitro* at a concentration of 1.35 mg/ml (Meyer et al., 1996).

Results of two previous studies conducted by Heyman et al. (2015) and Yazdi et al. (2019) on the metabolomic analysis of anti-HIV activity of *Helichrysum* species have been integrated in this study. This study therefore aimed to identify biomarkers for anti-HIV activity in the aqueous methanolic extracts of the aerial parts of 57 *Helichrysum* species by using NMR spectroscopy and multivariate modeling (PCA and OPLS-DA).

## Materials and Methods

### Plant Material

The aerial parts of 59 extracts of selected *Helichrysum* species (57 species, one variety and one subspecies, Table 1) were collected from different geographical regions in South Africa during spring and summer (Permit no: OP 4928/2010). Herbarium specimens were identified by taxonomists from the South African National Biodiversity Institute (SANBI) together with the personnel at the H.G.W.J. Schweickerdt Herbarium (University of Pretoria).

**TABLE 1:** Analysed *Helichrysum* species and their herbarium voucher numbers and anti-HIV screening results (% inhibition). No activity against HIV-1 was observed for the non-polar extracts at 2.5 μg/ml (Heyman et al., 2015; Yazdi et al., 2019).

Selected Plants	Abbreviation	PRU^a^	Location and GPS	%Inhibition		
		Voucher No.		25 μg/ml	2.5 μg/ml	25 μg/ml
				Polar	Polar	Non-polar
*H. acutatum* DC.	*H_acu*	121012, 117098	Limpopo, Tzaneen	CA	CA	CA
			23˚56′ S, 29˚56′ E			
*H. adenocarpum* DC.	*H_aden*	120986	Mpumalanga, Amsterdam	122	NO	NO
			26˚00′ S, 30˚00′ E			
*H. albilanatum* Hilliard	*H_albi*	121022	Mpumalanga, BKNR^b^	CA	85	CA
			25˚00′ S, 30˚00′ E			
*H. alliodes* Less	*H_all*	117113	KwaZulu-Natal, Drakensberg	NA	NA	CA
			28˚58′ S, 29˚26′ E			
*H. anomala* Less	*H_ano*	117112	KwaZulu-Natal, Drakensberg	NA	NA	95
			28˚58′ S, 29˚26′ E			
*H. appendiculatum* (L.f.) Less	*H_app*	117101	KwaZulu-Natal, Drakensberg	82	NA	NA
			28˚57′ S, 29˚12′ E			
*H. argyrophyllum* DC.	*H_argy*	120814	Western Cape, KBG^c^	CA	NO	CA
			33˚00′ S, 18˚00′ E			
*H. athrixiifolium* (Kuntze) Moeser	*H. athr*	121537	Gauteng, Pretoria	CA	NA	CA
			25˚00′ S, 28˚00′ E			
*H. aureonitens* Sch.Bip	*H_au1*	117111	KwaZulu-Natal, Drakensberg	NA	NA	88
			28˚58′ S, 29˚26′ E			
*H. aureum* (Houtt.) Merr	*H_ aure*	121002	Mpumalanga, BKNR^b^	CA	95	CA
			25˚00′ S, 30˚00′ E			
*H. aureum* var. *monocephalum* (DC.) Hilliard	*H_aure_mo*	121008	Mpumalanga, BKNR^b^	126	NO	CA
			25˚00′ S, 30˚00′ E			
*H. caespititium* (DC.) Harv	*H_ caes*	121538	Gauteng, Pretoria	124	NO	CA
			25˚00′ S, 28˚00′ E			
*H. callicomum* Harv	*H_call*	121005	Mpumalanga, BKNR^b^	CA	NO	108
			25˚00′ S, 30˚00′ E			
*H. cephaloideum* DC.	*H_cep*	121018, 117127	Mpumalanga, BKNR^b^	115	NO	108
			25˚00′ S, 30˚00′ E			
*H. chionosphaaerum* DC.	*H_chi*	117097	KwaZulu-Natal, Drakensberg	NA	NA	104
			28°58′ S, 29°26′E			
*H. chrysargyrum* Moeser	*H_chry*	121004	Mpumalanga, BKNR^b^	120	103	CA
			25˚00′ S, 30˚00′ E			
*H. confertum* N.E.Br	*H_con*	96720	Eastern Cape, Rhodes	NA	NA	CA
			30° 43′ S, 28° 08′ E			
*H. cymosum* (L.) D.Don subsp. *calvum* Hilliard	*H_ccl*	117142	KwaZulu-Natal, UNR^d^	70	NA	114
			31˚05′ S, 30˚17′ E			
*H. cymosum* (L.) D.Don subsp. *cymosum* (L.)D.Don	*H_ccy*	117120	KwaZulu-Natal, UNR^d^	75	NA	90
			31˚05′ S, 30˚17′ E			
*H. dasyanthum* (Willd.) Sweet	*H_dasy*	120813	Western Cape, KBG^c^	CA	110	CA
			33˚00′ S, 18˚00′ E			
*H. difficile* Hilliard	*H_dif*	117122	KwaZulu-Natal, Drakensberg	NA	NA	NA
			29˚03′ S, 29˚24′ E			
*H. drakensbergense* Killick	*H_dra*	117121	KwaZulu-Natal, Drakensberg	NA	NA	NA
			29˚03.68′ S, 29˚23.73′ E			
*H. gerberifolium* A.Rich	*H_gerb*	121003	Mpumalanga, BKNR^b^	CA	92	NO
			25˚00′ S, 30˚00′ E			
*H. harveyanum* Wild	*H_harv*	121547	Limpopo, Tzaneen	CA	107	CA
			23˚56′ S, 29˚56′ E			
*H. herbaceum* (Andrews) Sweet	*H_her*	117099	KwaZulu-Natal, Drakensberg	NA	NA	NA
			28˚57.82′ S, 29˚12.28′ E			
*H. kraussii* Sch.Bip	*H_krau*	121025	Pretoria, PNBG^e^	CA	97	125
			25˚44′ S, 28˚16′ E			
*H. lepidissimum* S.Moore	*H_lepi*	121009	Mpumalanga, BKNR^b^	CA	108	CA
			25˚00′ S, 30˚00′ E			
*H. mariepscopicum* Hilliard	*H_mari*	121013	Mpumalanga, BKNR^b^	121	NO	CA
			25˚00′ S, 30˚00′ E			
*H. melanacme* DC	*H_mel*	117107	KwaZulu-Natal, Drakensberg	NA	NA	84
			28˚56.98′ S, 29˚12.44′ E			
*H. miconiifolium* DC.	*H_mic*	117102	KwaZulu-Natal, Drakensberg	NA	NA	NA
			28˚57.82′ S, 29˚12.28′ E			
*H. milleri* Hilliard	*H_mill*	121015	Mpumalanga, BKNR^b^	133	NO	129
			25˚00′ S, 30˚00′ E			
*H. mimetes* S.Moore	*H_mime*	121017	Mpumalanga, BKNR^b^	132	121	CA
			25˚00′ S, 30˚00′ E			
*H. mundtii* Harv	*H_mund*	121014	Mpumalanga, BKNR^b^	CA	NO	CA
			25˚00′ S, 30˚00′ E			
*H. mutabile* Hilliard	*H_muta*	121021	Mpumalanga, BKNR^b^	CA	111	CA
			25˚00′ S, 30˚00′ E			
*H. natalitium* DC.	*H_nat*	117669	KwaZulu-Natal, Greytown	NA	NA	CA
			29˚24.35′ S, 30˚54.73′ E			
*H. nudifolium* (L.) Less. var. *nudifolium* (L.) Less	*H_nudi*	117104	Limpopo, Tzaneen	120	NO	CA
			23˚56′ S, 29˚56′ E			
*H. odorotassimum* (L.) Sweet	*H_odo*	117106	Gauteng, Pretoria	NA	NA	CA
			25˚00′ S, 28˚00′ E			
*H. opacum* Klatt	*H_opac*	121019	Mpumalanga, BKNR^b^	CA	100	CA
			25˚00′ S, 30˚00′ E			
*H. oreophilum* Klatt	*H_or-1*	117096	Limpopo, Tzaneen	NA	NA	112
			23˚56′ S, 29˚56′ E			
*H. oxyphyllum* DC.	*H_oxy*	117670	Limpopo, Tzaneen	102	NA	CA
			23˚56′ S, 29˚56′ E			
*H. pallidum* DC.	*H_pal*	117108	Limpopo, Tzaneen	NA	NA	96
			23˚56′ S, 29˚56′ E			
*H. panduratum* O. Hoffm	*H_pan*	117662	KwaZulu-Natal, New Hanover	NA	NA	NA
			29˚21′ S, 30˚32′ E			
*H. pannosum* DC.	*H_pann*	117144	KwaZulu-Natal, Ken Gaze`s Farm	NA	NA	NA
			31˚05.27′ S, 30˚17.90′ E			
*H. patulum* (L.) D.Don	*H_patu*	121536	Western Cape	CA	114	CA
			33˚55′ S, 18° 51′ E			
*H. petiolare* Hilliard and B.L.Burtt	*H_peti*	121535	Western Cape	CA	NO	CA
			33˚55′ S, 18° 51′ E			
*H. pilosellum* (L.f.) Less	*H_pil*	117110	Limpopo, Tzaneen	NA	NA	109
			23˚56′ S, 29˚56′ E			
*H. platypterum* DC.	*H_plat*	121011	Mpumalanga, BKNR^b^	CA	118	100
			25˚00′ S, 30˚00′ E			
*H. polycladum* Klatt	*H_poly*	121016	Mpumalanga, BKNR^b^	CA	NO	95
			25˚00′ S, 30˚00′ E			
*H. populifolium* DC.	*H_pop*	117138	KwaZulu-Natal, UNR^d^	84	NA	NA
			31˚95.83′ S, 30˚17.43′ E			
*H. reflexum* N.E.Br	*H_refl*	121006	Mpumalanga, BKNR^b^	CA	NO	CA
			25˚00′ S, 30˚00′ E			
*H. setosum* Harv	*H_seto*	121539	Gauteng, Pretoria	CA	81	103
			25˚00′ S, 28˚00′ E			
*H. splendidum* (Thunb.) Less	*H_spl-1*	117124	Limpopo, Tzaneen	NA	NA	85
			23˚56′ S, 29˚56′ E			
*H. subluteum* Burtt Davy	*H_sub*	117123	KwaZulu-Natal, Drakensberg	NA	NA	NA
			29˚03.71′ S, 29˚23.70′ E			
*H. sutherlandii* Harv	*H_sut*	117115	KwaZulu-Natal, Drakensberg	NA	NA	NA
			29˚03.71′ S, 29˚23.70′ E			
*H. truncatum* Burtt Davy	*H_trun*	121020	Mpumalanga, BKNR^b^	CA	98	CA
			28˚58.72′ S, 29˚13.80′ E			
*H. umbraculigerum* Less	*H_umb*	117100	KwaZulu-Natal, Drakensberg	NA	NA	109
			28˚57.82′ S, 29˚12.28′ E			
*H. vernum* Hilliard	*H_ver*	117116	KwaZulu-Natal, Drakensberg	NA	NA	CA
			28˚58.08′ S, 29˚14.11′ E			
*H. wilmsii* Moeser	*H_wilm*	121007	Mpumalanga, BKNR^b^	CA	118	CA
			25˚00′ S, 30˚00′ E			
*H. zeyheri* Less	*H_zeyh*	121534	Northern Cape, Kuruman	CA	105	85
			27˚11′ S, 23˚00′ E			

*CA, cytotoxic activity observed; NA, no activity; NO, not observable.*

a *H.G.W.J., schweikerdt herbarium of the university of pretoria.*

b *Buffelskloof Nature Reserve.*

c *Kirstenbosch Botanical Garden.*

d *Umtamvuna Nature Reserve*.

e *Pretoria National Botanical Garden*.

### Plant Extraction

All plants were dried in the dark at room temperature. Dried material (5 g) was ground into small pieces but not to a fine powder. Different solvent systems with increasing polarity [hexane, dichloromethane (DCM), acetone (Ace), and methanol (MeOH): water (50:50)] were used for extractions. Extraction of the collected plant material was done on a SpeedExtractor E-914/E-916 (Buchi, Switzerland) in 40 ml steel pressure vessels. Thereafter, the filtrate was concentrated under vacuum to dryness using a Genevac (EZ-2 Plus, GeneVac, United Kingdom) (Heyman et al., 2015; Yazdi et al., 2019).

### ^1^H NMR Analysis

A Varian 600 MHz spectrometer (Council for Scientific and Industrial Research, CSIR) was used for the ^1^H NMR analysis of the samples. The 12 mg of polar fractions were re-dissolved in 800 ml (15 mg/ml) in a buffered mixture of CD_3_OD and KH_2_PO_4_-D_2_O solution, with the pH adjusted to pH 6.0 with NaOD (1M). The internal standard trimethylsillyl propionic acid-D4 sodium salt (0.1% TSP- 0.00 ppm) was used for spectral referencing of the 50% methanolic samples. For each spectrum, 64 scans were recorded with a spectral width of 14 ppm. The temperature was kept at 25°C constantly. The total transients of the standard 1D spectra were set to 46 with a three-second relaxation delay, and the acquisition time for each transient scan set to 3 seconds. The magnet shimming was done automatically for optimal and consistent spectral resolution.

All ^1^H NMR spectra were referenced (based on residual CH_3_OH; δ3.310 ppm), baseline-corrected (Whittaker smoother), automatic phase corrected, and normalised by scaling the spectral intensities to 0.1% trimethylsilylpropanoic acid (TSP) using MestReNova 14.2 (Mestrelab Research S.L.). The region of 0.00-10.00 ppm was reduced to bins of 0.04 ppm in width. The regions ranging from 3.28 to 3.36 ppm (residual MeOH) and 4.60–5.00 ppm (residual water) were removed before statistical analysis. The ASCII files generated were then processed in Microsoft Excel and imported into SIMCA-P 14 (Umetrics, Umeå, Sweden). The data was Pareto scaled before being subjected to PCA and OPLS analyses.

### Anti-HIV Screening Activity

The procedures of anti-HIV screening and the colorimetric HIV-1 reverse transcriptase assay were done as previously described by Heyman et al. (2015) and Yazdi et al. (2019).

## Results

The results of the anti-HIV screening of the 59 extracts of selected *Helichrysum* species, are shown in Table 1, consistent with the results from previous studies (Heyman et al., 2015; Yazdi et al., 2019).

**FIGURE 1:**
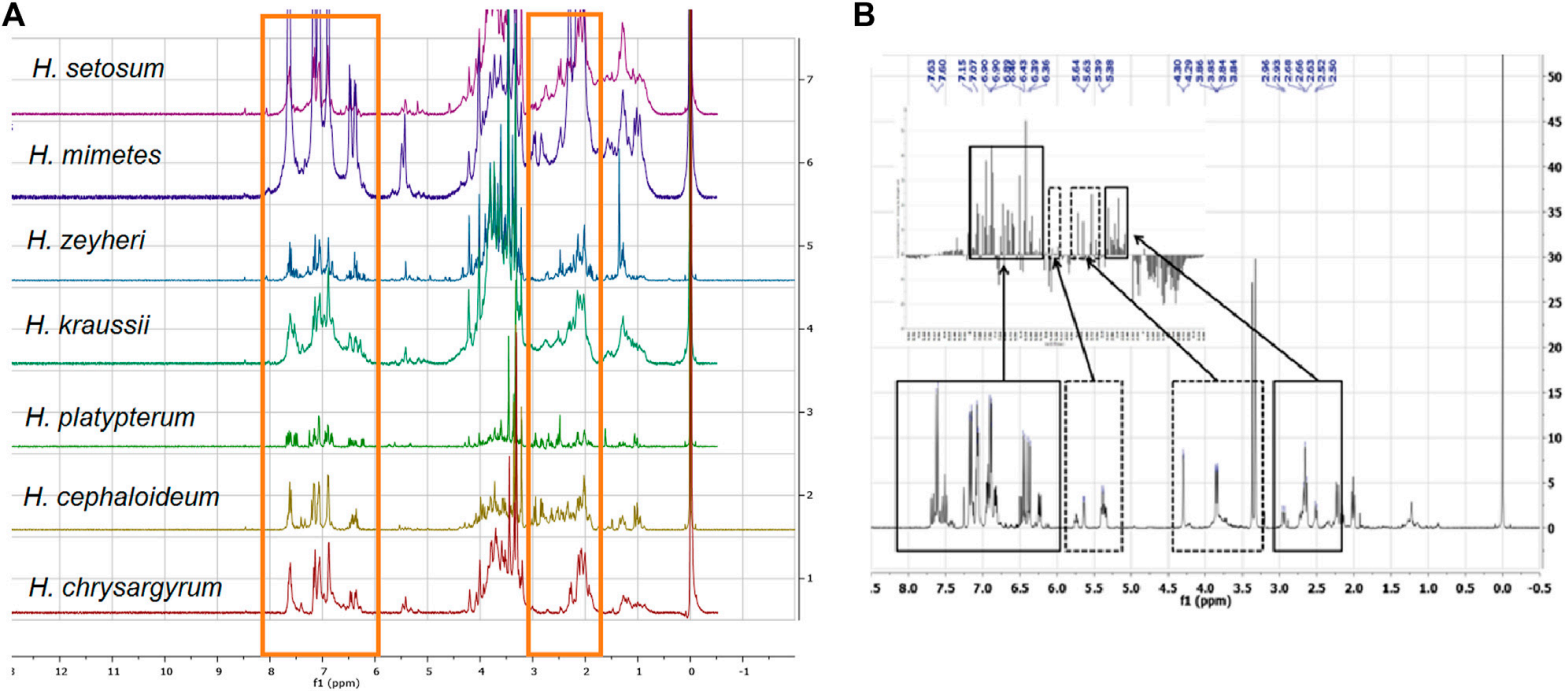
Comparison of the stacked ^1^H NMR spectra of the most active polar *Helichrysum* species extracts studied by Yazdi et al. (2019). **(A)**. with the NMR spectrum of a fraction isolated from *H. populifolium* with the best active profile from the study conducted by Heyman et al. (2015). **(B)**. The chemical shifts linked to the caffeoylquinic acid type compounds were present in the *Helichrysum* polar extracts (red boxes). **(A)**. and areas with the most contribution are highlighted with solid line boxes. **(B)**.

The proton NMR spectra of the *Helichrysum* species extracts with the highest activity against HIV (Table 1) showed significant similarities with the presence of aromatic compounds (6.50‒7.50 ppm) and carbohydrate moieties (1.80‒2.50 and 3.00‒4.10 ppm), characteristic signals of phenylpropanoids or chlorogenic acids (Figure 1). These compounds are a diverse family of organic compounds that are synthesized by plants from the amino acids, phenylalanine and tyrosine. Various bio-activities have been reported for these phytochemicals such as antiviral, anti-cancer and other biological effects (Miyamae et al., 2011).

One of the major types of compounds present in *Helichrysum* species are chlorogenic acids (Albayrak et al., 2010). The ^1^H NMR spectra of all fractions isolated in two previous studies conducted by Heyman et al. (2015) and Yazdi et al. (2019) showed that the anti-HIV compound(s) could probably be caffeoylquinic acids. Comparison of the stacked NMR spectra showed that all the chemical shifts linked to the caffeoylquinic acid type compounds existed in all *Helichrysum* species extracted (Figure 1).

**FIGURE 2:**
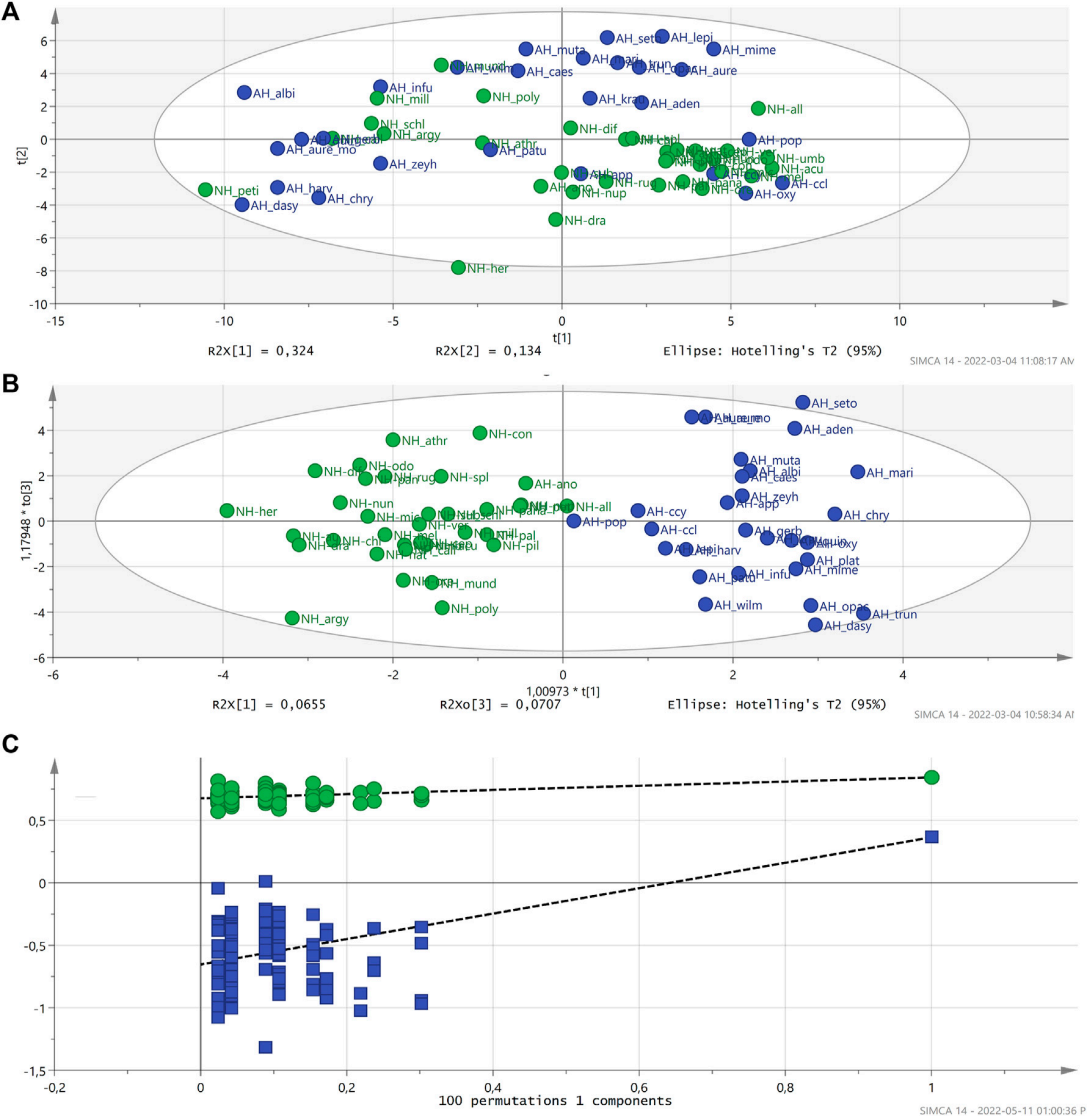
PCA score plot did not exhibit a significant correlation between active and non-active *Helichrysum* polar extracts. R2X: 0.766 and Q2 (cum): 0.571. 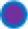
Active extracts, 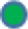
extracts with no activity. **(A)**. The OPLS-DA score plot showing good separation of the active and non-active *Helichrysum* polar extracts with some overlap in the center, R2X = 0.683 and Q2 (cum) = 0.227. 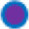
Active extracts, 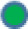
extracts with no activity. **(B)**. The OPLS-DA plots were validated by Permutation (100 permutations on the first five components). 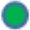
R2, 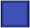
Q2. **(C)**.

All polar extracts were subjected to metabolomic analysis. To fast-track the selection of the possible biomarkers, metabolomic tools were used to investigate similarities and differences in the chemical profiles of the extracts of the 57 *Helichrysum* species and one subspecies and one variant using ^1^H NMR spectroscopy (Supplementary Material 1). Since all samples belonged to the *Helichrysum* genus, it was predicted that not many different groups would be obtained in the PCA. The datasets used for the PCA score plots did not show distinct grouping correlating with the activity of the extracts. The PCA model with R2X = 0.766 and Q2 = 0.571 values for component 2 indicated good predictability and reliability of the model (Figure 2).

In the OPLS-DA analysis (Figure 2), anti-HIV activity data was included as a secondary observation, which assisted in the correlation of the phytochemical composition and biological activity of the samples. The extent of grouping on similarity in chemical composition and bioactivity was satisfactory in this analysis. It indicates that, based on the biological activity of polar extracts, there is a distinct phytochemical difference between these two groups of extracts (active and non-active). The variation in X explains much of the variation with the R2X (cumulative) being 75%. The predictive component (P1) only explained 4.6% of the variation in X related to the separation of the samples based on the activity. Although, the description of the variation in the samples was acceptable the predictability of the model was slightly lower with R2X = 0.683, R2Y = 0.843 and Q2 (cum) = 0.227. Based on the Q2 of approximately 0.25, the predictability of the model was not significant but acceptable (Eriksson et al., 2013; Wu and Wang, 2015). The OPLS-DA plots were generated with a Hotelling’s T2 test of a 95% significance, validated by Permutation (100 permutations on the first five components) (Figure 2) and subjected to cross validated (CV)-ANOVA significance testing (*p*-value < 0.05).

**FIGURE 3:**
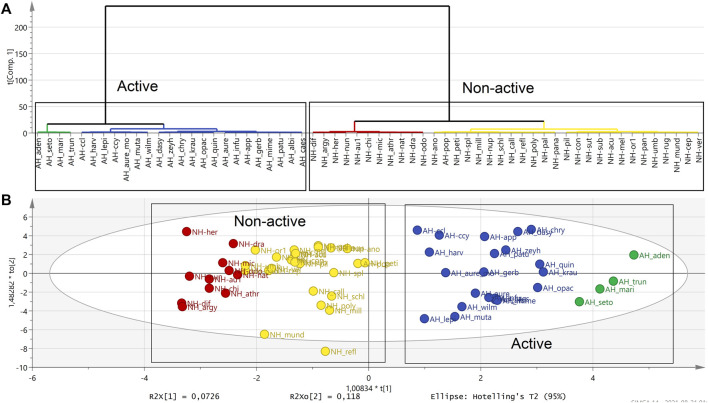
HCA dendrogram showing the attribute distances between each group of sequentially merged classes in active (blue and green) and non-active polar extracts (red and yellow). **(A)**. Clustering observed in the OPLS-DA score scatter plot supports the HCA dendrogram analysis by separating active and non-active *Helichrysum* polar extracts. **(B)**. (AH: active *Helichrysum* species, NH: non-active *Helichrysum*).

Hierarchical cluster analysis (HCA), as a complementary data reduction and pattern recognition method, was used for finding the underlying structure of objects through a repetitive process that associates or dissociates object by object until all are equally and completely processed. The HCA showed separated groups in the OPLS-DA analysis in the active and also in non-active extracts (Figures 3A,B).

**FIGURE 4:**
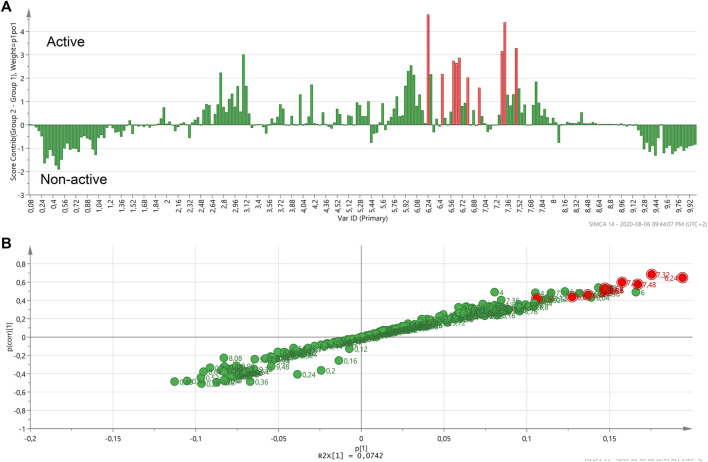
Contribution plot **(A)** and S-plot **(B)** generated two groups of the most active and non-active *Helichrysum* extracts and showing the typical signals of cinnamoyl units. In the contribution plot, upward bars indicate the NMR regions associated with the active samples, and downward bars indicate the NMR regions associated with the non-active samples. 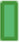, 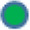
Buckets of whole NMR signals analysis. 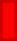, 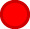
Potential biomarker NMR buckets.

An S-plot was generated based on the OPLS-DA score plot of the most active *Helichrysum* species (*H. mimetes*, *H. populifolium*, *H. platypterum*, and *H. symosum*) and selected the most non-active extracts. The S-plot clearly indicated the typical signals of cinnamoyl units 6.2 to 6.5 and 7.5–7.7 ppm in the active samples (Figure 4). It showed that these signals are the main correlate with for the anti-HIV activity of active *Helichrysum* species.

**FIGURE 5:**
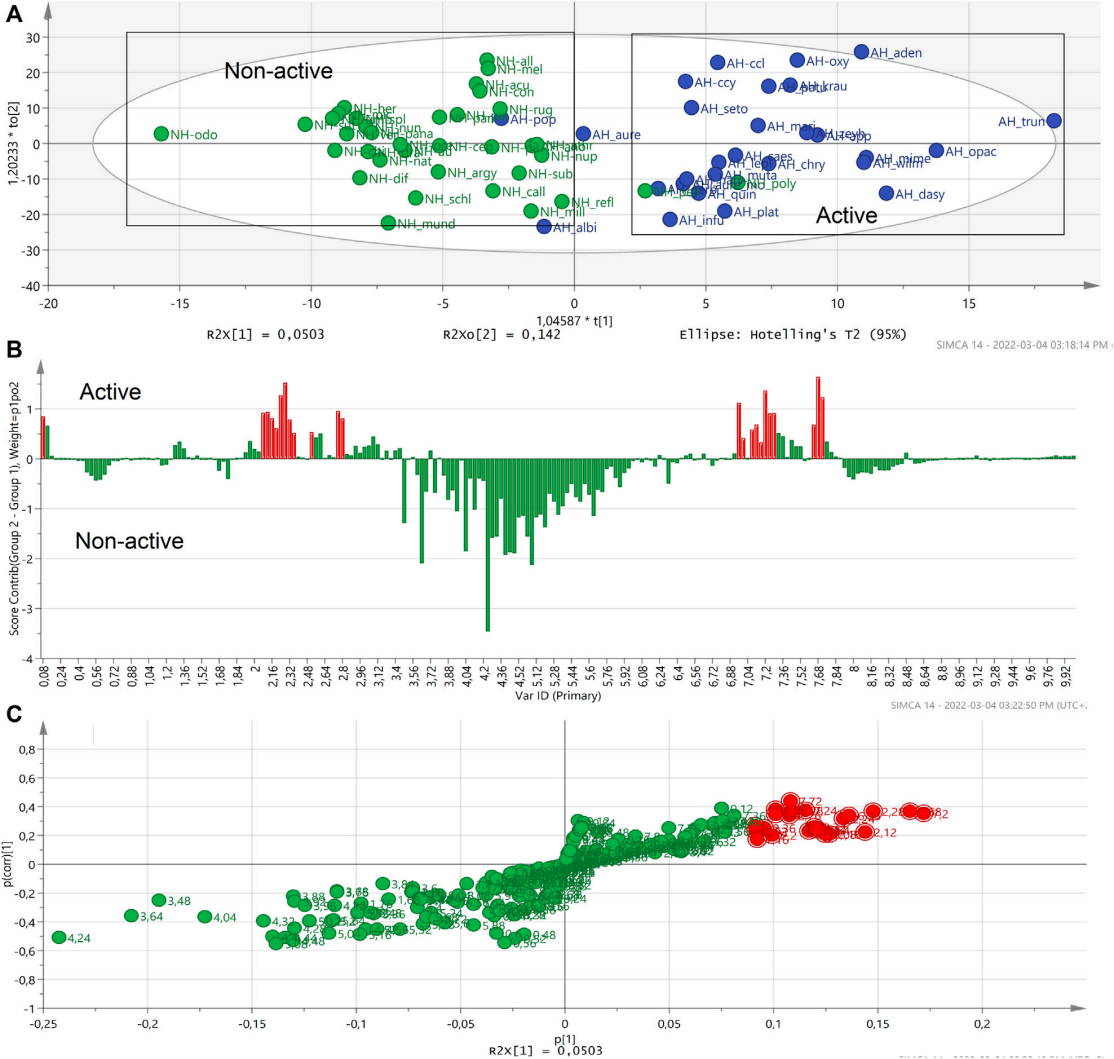
The OPLS-DA score plot of the selected active and selected non-active *Helichrysum* polar extracts. **(A)**. The contribution plot generated by comparing two groups of active and non-active *Helichrysum* extracts and showing the typical signals of CQA units. In the contribution plot, upward bars indicate the NMR regions associated with the active samples, and downward bars indicate the NMR regions associated with the non-active samples **(B)**, the loading S-plot indicating the buckets that are most responsible with activity of the active *Helichrysum* extracts. **(C)**. 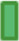, 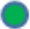
Buckets of NMR signals. 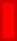, 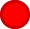
potential biomarkers NMR buckets.

A second contribution plot and S-plot (Figures 5B,C) were generated based on the OPLS score plot of selected active and selected non-active groups (Figure 5A). The generated S-plot indicated that the typical signals of the NMR chemical shifts associated with caffeoylquinic acids (CQA): mainly between 2.10–3.10 ppm and 6.20‒8.00 ppm (Figures 5B,C). It revealed that these signals correlate with the anti-HIV-1 activity of *Helichrysum* species.

**FIGURE 6:**
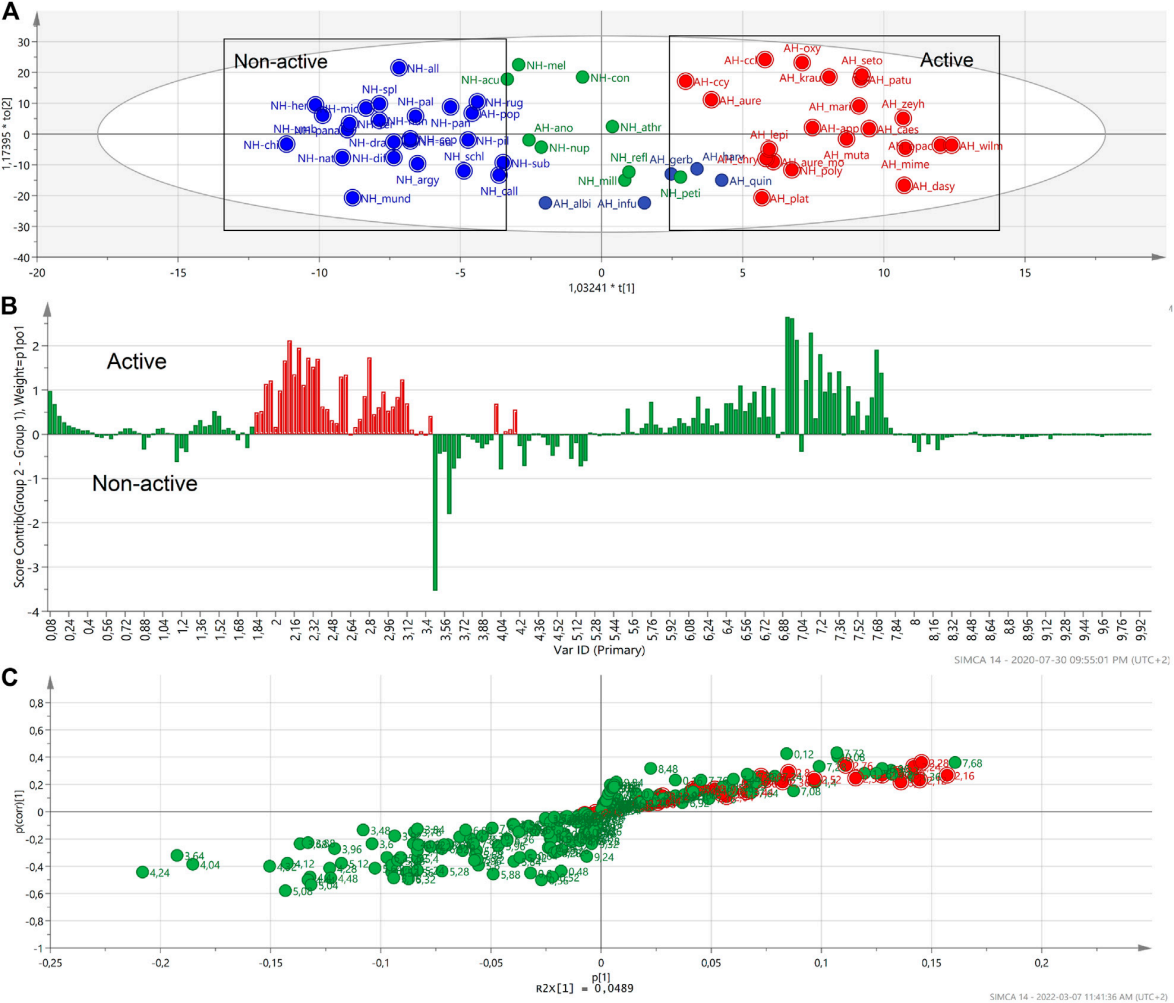
The OPLS-DA score plot of the active and non-active *Helichrysum* polar extracts. **(A)**. The red marked upward bars of the contribution plot and red spots on the S-plot generated by comparing active and non-active groups of *Helichrysum* extracts show the typical signals of quinic acid units in the active group. **(B,C)**. 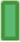, 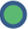
Buckets of NMR signals analysis. 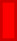, 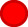
Potential biomarker NMR peaks.

Contribution and S-plots (Figures 6B,C) were generated based on the OPLS-DA score plot (Figure 6A) of active and a non-active samples identified by the HCA dendrogram. *H. odoratissimum*, *H. sutherlandii*, *H. oreophilum* from the non-active region and *H. adenocarpum* and *H. truncatum* from the active region were excluded as outliers to generate this OPLS-DA. Although the presence of CQA peaks is dominant in both plots, quinic acid peaks can also be seen. The generated profile could analyse the specific regions that can be related to the activity of the extract(s). The two plots indicated that the typical signals of quinic acid units: 1.85 to 2.20 and 3.35–4.20 ppm (Figure 6) are contributing to some of the anti-HIV activity. It was also previously reported that these signals are responsible for the activity of the anti-HIV RT of *H. mimetes* (Yazdi et al., 2019). It seemed that other types of compounds like sugars or amino acids did not play a major role in the activity of the *Helichrysum* species against HIV RT showing negative contribution for these regions on the contribution plot (Figure 6B). However, it must be considered that the differences in the chemical shifts of the bars above the line and bars below the line could be related to the concentration of the compounds in the two groups of extracts and not a presence or absence of compounds.

All data were analysed statistically of both bioactivity and metabolomic analyses for a better understanding of the anti-HIV activity of *Helichrysum* species and the *p*-value of the investigated models was significant (<0.05).

## Discussion

Heyman et al. (2015) identified five major compounds observed in the most active fraction six isolated from *H. populifolium*. The fractions were identified as being chlorogenic acid derivatives using LC-IT-TOF. The fraction patterns showed that three of the major compounds are dicaffeoylquinic acid (DCQA) that is 3,4-DCQA (516 Mr), 3,5-DCQA (516 Mr) and 4,5-DCQA (516 Mr). Two other types of chlorogenic type of compounds were identified by Heyman et al. (2015), tricaffeoylquinic acid (TCQA), 1,3,5-TCQA (678 Mr) and 5-malonyl-1,3,4-TCQA (764 Mr) (Supplementary Material 2A).

In the Yazdi et al. (2019) study, the phytochemical fingerprint of sub-fraction 15 isolated from *H. mimetes* and standard quinic acid (1,3,4,5-tetrahydroxycyclohexane carboxylic acid), were compared using UPLC-MS to confirm the identity of the compound as quinic acid. The mass spectrum in negative ionization mode is shown in Supplementary Material 2B. A reverse phase C18 column was used with MeOH:H_2_O as mobile phase. An elemental composition report of the MS analysis confirmed the presence of quinic acid in sub-fraction with the mass of 191.0564 and molecular formula of C_7_H_11_O_6_ (Supplementary Material 2B). The mass spectrum of the contaminant was confirmed as a sodium salt of formic acid.

This study on the data integration of two previous related studies (Heyman et al., 2015; Yazdi et al., 2019) on the chemistry and anti-HIV-1 activity of *Helichrysum* species showed that the chlorogenic acid type compounds (e.g. caffeoylquinic acids, cinnamoyl groups) and quinic acid, a building block of chlorogenic acids, can serve as biomarkers for anti-HIV activity. There are previous reports that showed the activity of CQA types of compounds against HIV integrase (HIV IN) (McDougall et al., 1998; Tamura et al., 2006). The study of Heyman (Heyman, 2013), also reported anti-HIV IN activity of 3,4-DCQA,3,5-DCQA and 4,5-DCQA at 0.71, 0.66 and 0.30 µM (Heyman, 2013). The obtained results are supported by McDugall et al. (1998) and Tamura et al. (2006) that indicated significant activity of several DCQA against HIV IN, ranging between 2 and 12 µM. There are no previous reports on anti-HIV RT activity of quinic acid apart from that of Yazdi et al. (2019) who isolated it from *H. mimetes* and showed promising anti-RT activity (IC_50_ = 53.82 μg/ml) comparable to the positive drug control, doxorubicin (IC_50_ = 40.31 μg/ml). Also, the molecular docking study on isolated quinic acid from *H. mimetes*, indicated that the quinic acid–RT complex showed good stability and good H-bonding (Yazdi et al., 2019). Thus, activity-based metabolomic studies on the chemistry and bioactivity of plants represent a unique approach to identify lead compounds without the need for extensive bioassay-guided fractionation. This may also lead to lower input cost and time to discover new therapeutics for various diseases.

## Data Availability

The original contributions presented in the study are included in the article/Supplementary Material, further inquiries can be directed to the corresponding author.
