# Supplementary material for: Identification of Anti-HIV Biomarkers of Helichrysum Species by NMR-Based Metabolomic Analysis
Source: Front Pharmacol. 2022 Jul 22;13:904231. doi: 10.3389/fphar.2022.904231 (PMC9355245; doi:10.3389/fphar.2022.904231)
Supplement: Supplementary file 1 [file Image1.pdf]

Supplementary material 2

A

MS^4 fragmentation data for putatively identified di- and tricaffeoylquinic acids from fraction 6 of *H. populifolium* (Heyman, 2013).

Compound	Parent ion m/z	MS^2 base peak m/z	MS^2 secondary ions										MS^3 base peak m/z	MS^3 secondary ions								MS^4 base peak m/z	MS^4 secondary ions							
			m/z	int*	m/z	int*	m/z	int*	m/z	int*	m/z	int*		m/z	int*	m/z	int*	m/z	int*	m/z	int*		m/z	int*	m/z	int*	m/z	int*	m/z	int*
3,4-DCQA	515.1	353.1	335.1	8	317.1	4	299.1	7			203.0	10	173.0	191.1	48	179.0	57	135.0	4			93.0	111.0	22						
3,5-DCQA	515.1	353.1											191.1	179.0	44	173.0	6	135.0	8			85.0	173.0	15	127.0	73	111.1	18	93.0	21
4,5-DCQA	515.1	353.1			317.1	12	299.1	13	255.1	2	203.0	17	173.0	191.1	35	179.0	57	135.0	100			93.0	173.0	53	127.0	60	111.1	66		
1,3,5-TCQA	677.1	497.1	515.1	21	469.1	3	353.1	21	335.1	16			335.1	179.0	6							173.0	179.0	30	161.0	12	135.0	12		
5-malonyl-1,3,4-TCQA or 3-malonyl-1,4,5-TCQA	763.1	497.1	515.1	60	469.1	24	353.1	5	335.1	2	317.1	7	353.1	335.1	4	299.1	8	255.1	2	203.0	12	173.0	191.1	30	179.0	39	135.0	7		

*Intensity

B

Chromatogram in negative mode of sub-fraction isolated from *H. mimetes* and standard quinic acid (molecular weight: 192.167 g/mol). (a) 0.69; 191.0562, (b) 0.75; 112.9852, (c) 0.60; 112.9852/ 1.17; 96.9596 (d) 0.56; 112.9852/ 0.75; 191.0565 (A). MS spectra in negative mode of sub-fraction isolated from *H. mimetes* and standard quinic acid. The extra peak at 405.1006 is because two quinic acid molecules linked with a sodium molecule (B) (Yazdi et al., 2019).

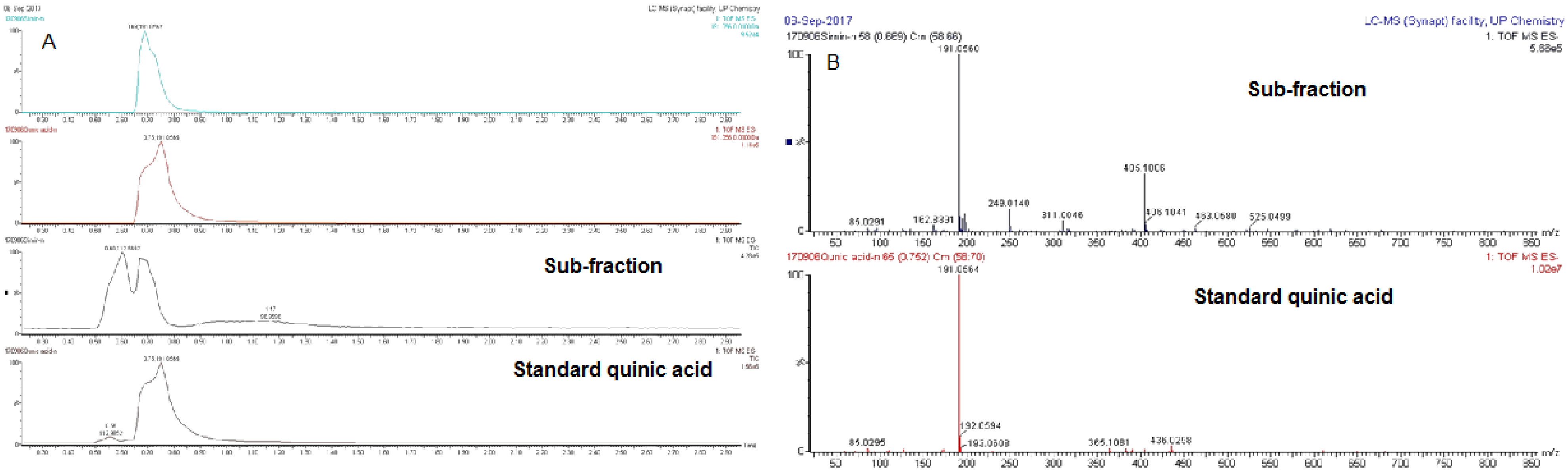

References

- Heyman, H. M. (2013). Identification of anti-HIV compounds in *Helichrysum* species (Asteraceae) by means of NMR-based metabolomic guided fractionation. [dissertation’s thesis]. University of Pretoria. http://hdl.handle.net/2263/40273
- Yazdi, S. E., Prinsloo, G., Heyman, H. M., Oosthuizen, C. B., Klimkait, T., and Meyer, J. J. M. (2019). Anti-HIV-1 activity of quinic acid isolated from *Helichrysum mimetes* using NMR-based metabolomics and computational analysis. *S. Afr. J. Bot.* 126, 328-339. doi: 10.1016/j.sajb.2019.04.023
